# A 1T2C FeCAP-Based In-Situ Bitwise X(N)OR Logic Operation with Two-Step Write-Back Circuit for Accelerating Compute-In-Memory

**DOI:** 10.3390/mi12040385

**Published:** 2021-04-01

**Authors:** Qiao Wang, Donglin Zhang, Yulin Zhao, Chao Liu, Qiao Hu, Xuanzhi Liu, Jianguo Yang, Hangbing Lv

**Affiliations:** 1 Institute of Microelectronics of Chinese Academy of Sciences, Beijing 100029, China; wangqiao@ime.ac.cn (Q.W.); zhangdonglin20@mails.ucas.ac.cn (D.Z.); zhaoyulin@ime.ac.cn (Y.Z.); liuchao2019@ime.ac.cn (C.L.); qhu@mail.ustc.edu.cn (Q.H.); xuanzhi@mail.ustc.edu.cn (X.L.); lvhangbing@ime.ac.cn (H.L.); 2 School of Microelectronics, University of Chinese Academy of Sciences, Beijing 100049, China; 3 School of Microelectronics, University of Science and Technology of China, Hefei 230026, China; 4 Zhejiang Lab, Hangzhou 311121, China

**Keywords:** ferroelectric capacitor, 1T2C, X(N)OR logic operation, nondestructive reading

## Abstract

Ferroelectric capacitors (FeCAPs) with high process compatibility, high reliability, ultra-low programming current and fast operation speed are promising candidates to traditional volatile and nonvolatile memory. In addition, they have great potential in the fields of storage, computing, and memory logic. Nevertheless, effective methods to realize logic and memory in FeCAP devices are still lacking. This study proposes a 1T2C FeCAP-based in situ bitwise X(N)OR logic based on a charge-sharing function. First, using the 1T2C structure and a two-step write-back circuit, the nondestructive reading is realized with less complexity than the previous work. Second, a method of two-line activation is used during the operation of X(N)OR. The verification results show that the speed, area and power consumption of the proposed 1T2C FeCAP-based bitwise logic operations are significantly improved.

## 1. Introduction

Von Neumann architecture is widely used in computer systems, where memory and computing are completely isolated. However, the memory access speed is much slower than the processor’s processing speed [1,2], which has become a “memory wall” that limits the computer’s overall performance. Recently, processing-in-memory (PIM) architecture has been proposed because of its potential to overcome the “memory wall” problem [3,4,5]. PIM stores operands through a memory array and calculates them in memory, reducing power consumption during memory access and data handling. Therefore, PIM has great potential in graph computing, speech processing, memory database, and real-time analysis [6]. Based on the different types of memory, the mainstream research of the PIM architecture is divided into two: one is volatile memory-based PIM, and the other is nonvolatile memory-based PIM [7,8]. Characteristics, such as low storage density, high-energy consumption, and latency of static random-access memory (SRAM) caused by the serial row-by-row access mode, makes SRAM-based PIM unsuitable for large and complex computing tasks [9,10]. Dynamic random-access memory (DRAM)-based PIM has higher array efficiency, but DRAM reading is a destructive operation [11,12]. Moreover, the data stored in volatile memory will disappear once the power down. Traditional nonvolatile memory (flash) has high storage density, low cost, and can achieve high-precision volume production operations. These advantages make flash very suitable for PIM [13]. However, flash is programmed only in blocks, and hence its performance under advanced technology is poor [14,15]. Specifically, SRAM memory cells that support in-memory X(N)OR operations usually adopt a 6T or 8T structure on memory arrays, which leads to poor memory efficiency [16]. Regular refresh operations are required in DRAM to maintain data, which leads to poor power consumption performance of DRAM-based in-memory X(N)OR logic. To deal with these issues, emerging nonvolatile memories with a high-speed of reading and writing, high density, low power consumption, and easy scaling have attracted more attention in recent years [17,18,19]. Among these, resistive random-access memory (RRAM) uses bipolar and unipolar memristors to realize the in-memory X(N)OR logic [20]. However, this method is accompanied by a complex manufacturing process, expensive preparation cost and extra peripheral circuits. Moreover, the analog current summation is usually adopted by RRAM and magnetoresistive random-access memory (MRAM) to effectively realize the in-memory X(N)OR logic operations [19,21,22], which leads to higher power consumption. However, FeRAM uses the charge-sharing function of FeCAPs to realize the X(N)OR logic to reduce power consumption. In addition, the preparation process of FeRAM memory cells (1T1C, 1T2C, or 2T2C) is simple and fully compatible with the logic process. Hence, FeCAPs with high process compatibility, high reliability, ultra-low programming current, and fast operation speed are promising candidates for the traditional volatile and nonvolatile memories.

Recently, the 1T1C-FeCAP-based PIM structure has been studied [23]. Due to the destructive-reading of FeCAPs, a complex write-back circuit is needed, which leads to the complexity of the design and an increase in power consumption and cost. In this study, a 1T2C FeCAP-based in situ bitwise X(N)OR logic operation scheme was proposed. A two-line activation technique was also adopted during the process of X(N)OR. Assistant circuits, including a sense amplifier (SA) and a control module, were designed to implement bit-by-bit X(N)OR operations in the same bit line (BL).

The 1T2C cell structure can overcome the destructive reading issue of the 1T1C FeCAP cell using a two-step write-back circuit. During the writing phase, the data are written in the two FeCAPs simultaneously. In the sensing phase, the charge of one FeCAP is read out, and the other is responsible for storing data and assisting in data rewriting.

## 2. Previous Related Studies

X(N)OR is a function that cannot be realized quickly and efficiently using traditional central processing unit (CPU)-based methods. However, the X(N)OR operation is an important logic operation, which has many important applications. Implementing X(N)OR effectively and cheaply has become a hot research topic.

**Figure 1:**
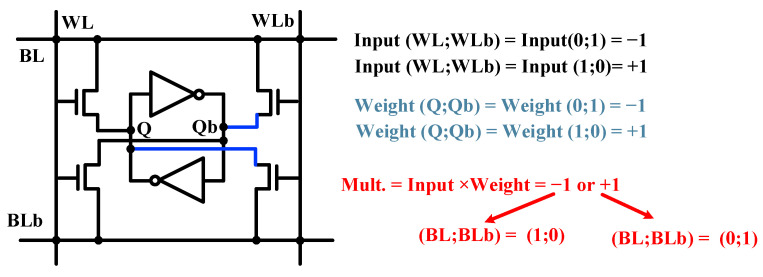
8T static random-access memory (SRAM) cell design approaches for in-memory X(N)OR logic operation.

SRAM-based in-memory-X(N)OR operation is difficult to be realized by 6T-type SRAM cell. Hence, an SRAM cell with an 8T structure [24] or even 12T structure [25] has been proposed to realize X(N)OR logic operations in SRAM. The operation principle of an 8T SRAM cell is shown in Figure 1. The 8T-type cell has two pairs of switch transistors controlled by a word line (WL) and word-line bar (WLb), respectively. A pair of switch transistors controlled by WL connect Q and Qb to bitline (BL) and bitline-bar (BLb). Moreover, a pair of switch transistors controlled by WLb connect Q and Qb to BLb and BL. Data stored in Q and Qb nodes is the weight of the X(N)OR logic. The voltages of WL and WLb are inputs of the X(N)OR logic. The output of the X(N)OR logic is the multiplication result of weight and input, as shown in Figure 1. Finally, X(N)OR operation can be realized by using an 8T SRAM cell. However, due to the additional transistors and metal routing [24], the cell size of an 8T SRAM cell is much larger than that of a conventional 6T SRAM cell and other memory cells.

**Figure 2:**
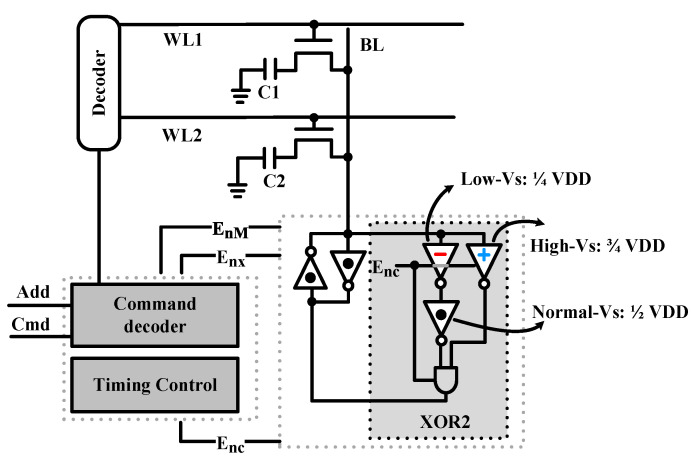
The reconfigurable sense amplifier is used to implement logic operations. Reprinted with permission from ref. [26]. Copyright @ 2019 IEEE.

In the work of Angizi and Fan [26], a DRAM-based in-memory XOR2 circuit was designed. The DRAM memory cell is the basic computing cell. To realize the XOR2 logic, they proposed a new reconfigurable SA, as shown in Figure 2. The XOR2 circuit consists of three inverters, having different switching voltages (Vs), and an AND gate. Then, the SA can distinguish “00”, “01” and “11” states. The Low-Vs (low switching voltage) inverter uses 1/4 VDD as the switching voltage to realize the NOR2 function. Simultaneously, the High-Vs (high switching voltage) inverter uses 3/4 VDD as the switching voltage to realize the NAND2 function. Finally, XOR2 logic can be realized after a CMOS AND gate in a single memory cycle. Moreover, to realize the XOR2 function, two capacitors connected to the same BL are read out simultaneously, and charge-sharing is implemented on the BL to implement logical operations. The main disadvantages of this method are the large area of the XOR2 circuit and the volatility of DRAM cells that need a periodic refresh.

**Figure 3:**
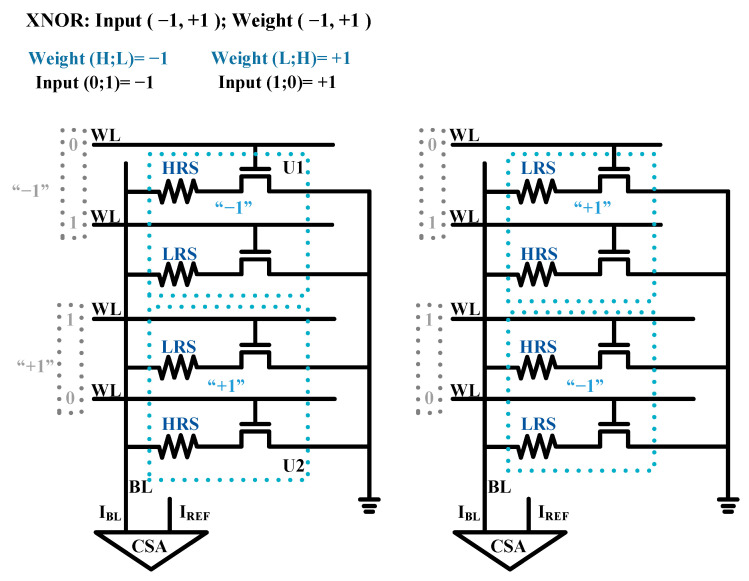
The sequential bit-cell design for X(N)OR implementation. Reprinted with permission from ref. [20]. Copyright @ 2018 IEEE.

Moreover, Xiaoyu Sun [20] and our previous study [27] proposed the in-memory X(N)OR logic operations based on novel nonvolatile memory (NVM). In the work of Xiaoyu Sun, the sequential X(N)OR-RRAM architecture was proposed, as shown in Figure 3. The calculating units U1 and U2 represent the weights “−1” and “1”, respectively. For the input of X(N)OR logic, the two WLs of a calculating unit are in a complimentary state where (0, 1) represents “−1” and (1, 0) represents “+1”. In this method, the value of the current that flows through each calculating unit during readout is dependent on the multiplication result of its input and weight. However, the analog current summation is usually adopted by RRAM to effectively realize the in-memory X(N)OR logic, which leads to higher power consumption.

**Figure 4:**
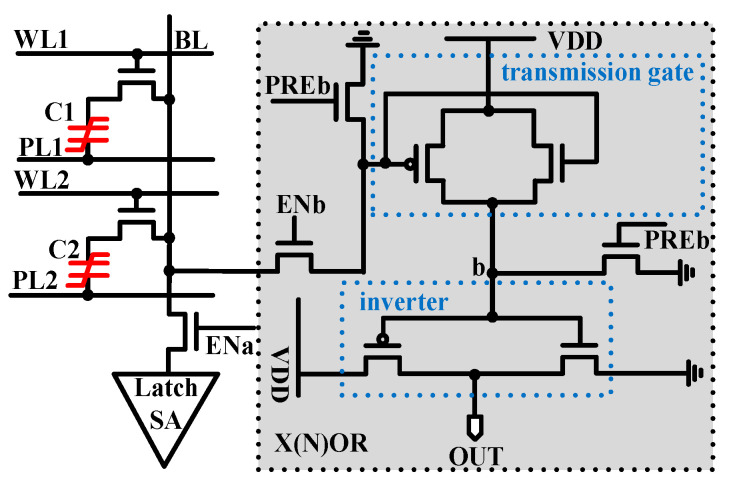
The X(N)OR circuit is based on the 1T1C-type ferroelectric capacitor (FeCAP) [27].

In our previous study, the X(N)OR operation based on the 1T1C FeCAP was proposed, as shown in Figure 4. Six MOS transistors were used in the circuit to achieve the X(N)OR operation. Two rows of data stored on the same BL are simultaneously read out, and then the charge-sharing function is realized. The signal of the transmission gate, which is triggered by the charge-sharing results of the BL voltage, is used to realize the X(N)OR operation.

Nevertheless, since the reading of the FeCAP is destructive and complex write-back circuits are needed to prevent data loss, the complexity of the design and the cost and power consumption are also increased.

The two representative studies are an X(N)OR logic function based on DRAM that was proposed by Angizi and Fan [26] and an X(N)OR logic function based on the 1T1C FeCAP that was proposed by our previous work [27].

## 3. Proposed 1T2C FeCAP-Based X(N)OR Logic Operation

Compared with the work of Angizi and Fan, we proposed a 1T2C FeCAP-based X(N)OR logic operation circuit that has lower power consumption and a smaller area. To reduce the complexity of the design of the write-back circuit, a two-step write-back circuit was designed that fully utilized the advantages of the 1T2C cell.

### 3.1. Operation of the Proposed 1T2C Cell

**Figure 5:**
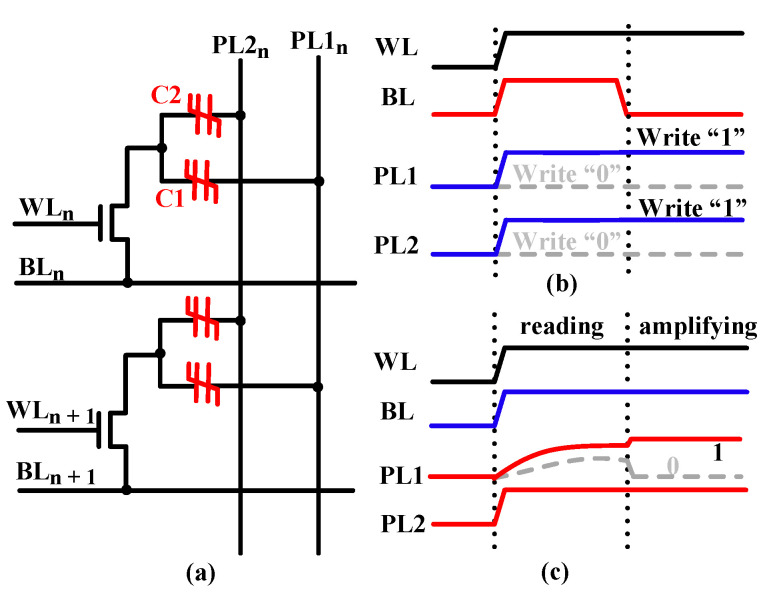
(**a**) A 2 × 1 array of the 1T2C cell. (**b**) Write timing. (**c**) Read timing.

In the 1T2C cell, a transistor is used to select the two FeCAPs, as shown in Figure 5a. The write and read timings of the 1T2C cell are shown in Figure 5b,c, respectively.

During writing, the same voltage pulse is applied to the plate lines, PL1 and PL2, to polarize the two FeCAPs to the same state. During reading the 1T1C FeCAP cell, PL is applied with a reading pulse, and BL is left floating [28,29]. Then, BL is charged by the polarized charge in the FeCAP. Finally, the BL voltage is compared with the reference voltage using SA to read out the stored data. For 1T2C, when the FeCAP C1 is read, the polarization state of the C2 will be affected by the BL voltage. Hence, during the reading process of 1T2C, BL and PL2 are applied with the same read pulse, and PL1 is left floating. Then, PL1 is charged by the polarized charge in the FeCAP, that is, C1. Finally, the PL1 voltage is compared with the reference voltage using SA to read out the stored data.

**Figure 6:**
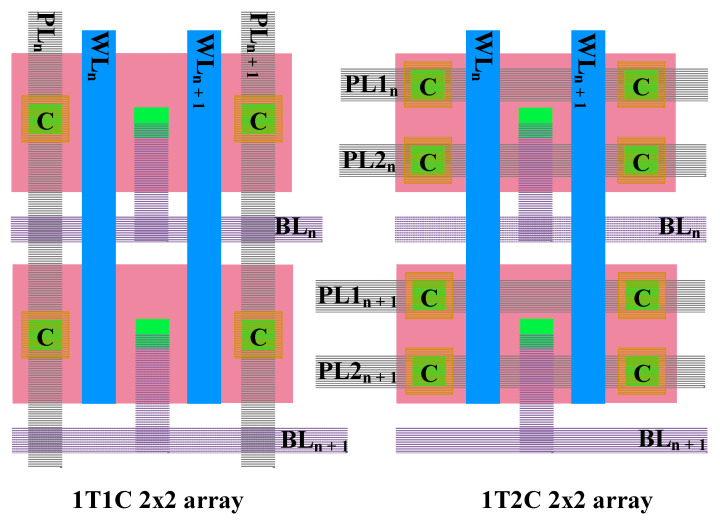
Layouts of the 1T1C and 1T2C FeCAP arrays.

The 1T2C-type FeCAP has only little effect on the area of the memory cell because the area of the transistor determines the cell area. The area of the transistor is substantially much larger than that of the FeCAP, as shown in Figure 6.

### 3.2. Dual-Row In-Memory X(N)OR Operation

**Figure 7:**
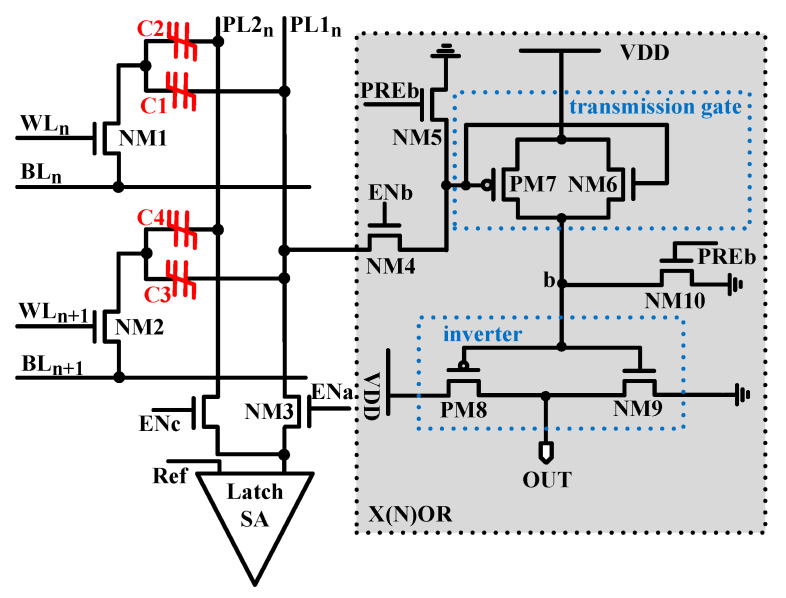
Schematic of the proposed mode switchable sense amplifier (SA).

X(N)OR and addition functions are prerequisites for accelerating various applications [30,31]. To realize the X(N)OR operation in the proposed 1T2C FeCAP, a mode-switchable SA circuit is designed, as shown in Figure 7.

The X(N)OR circuit consists of a latch SA, an inverter, and a transmission gate. The working mode of the SA circuit is changed by using two transistors NM3 and NM4. The working mode of the proposed SA is switched to traditional SA when NM4 is turned off, and NM3 is turned on. Conversely, the work mode of the proposed SA is switched to X(N)OR operation when NM3 is turned off, and NM4 is turned on.

**Figure 8:**
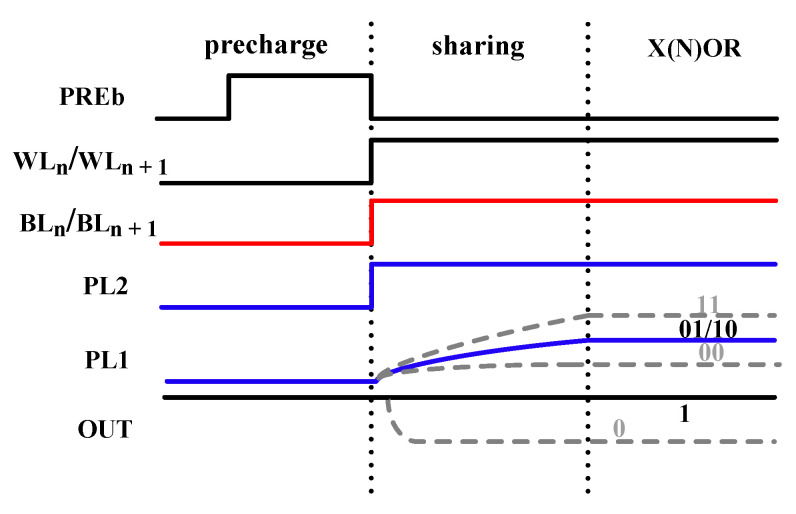
Timing of X(N)OR.

As shown in Figure 8, the X(N)OR operation is divided into three phases. In the precharging phase, the residual charge on PL1 and node b is released by the two precharge transistors NM5 and NM10. In the charge-sharing phase, the charges in C1 and C3 are dashed out. Meanwhile, the amount of charges of C2 and C4 remains constant. Then, PL1 is charged using the function of charge-sharing of the two FeCAPs, C1 and C3. In the X(N)OR phase, the on–off states of transistors PM7 and NM6 are determined by the voltage of PL1(VPL1) to realize X(N)OR.

When the data in C1 and C3 is “00”, PL1 is charged to a lower voltage. Consequently, PM7 is turned on, and NM6 is turned off. Then the voltage of node b is increased to VDD. Finally, the output of the X(N)OR circuit is “0”. When the stored data in C1 and C3 is “01” or “10”, PL1 is charged to a medium voltage. Consequently, PM7 and NM6 are turned off. Then the voltage of node b remains “0”. Finally, the output of the X(N)OR circuit is “1”. When the stored data in C1 and C3 is “11”, PL1 is charged to a higher voltage. Consequently, NM6 is turned on, and PM7 is turned off. Then the voltage of node b is increased to VDD-Vth6, where Vth6 is the threshold voltage of node NM6. Finally, the output of the X(N)OR circuit is “0”. Through these operations, the X(N)OR logic operation is realized.

### 3.3. Two-Step Write-Back Circuit

**Figure 9:**
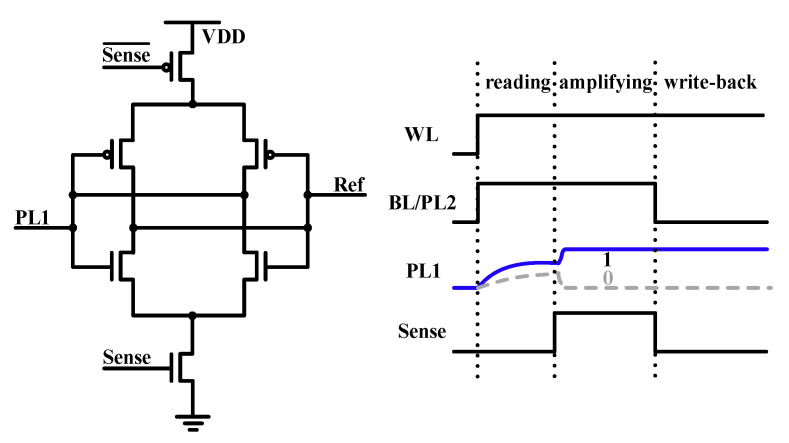
Schematic and timing of the latch SA.

Since the reading of the FeCAP is destructive, the stored data needs to be rewritten after the read operation [32,33]. For the traditional SA mode, the output of the SA will be fed back to the memory cell. As shown in Figure 9, when the read pulse on BL is pulled down, the rewriting of the data in the FeCAP is completed with the assistance of the latch SA [34].

**Figure 10:**
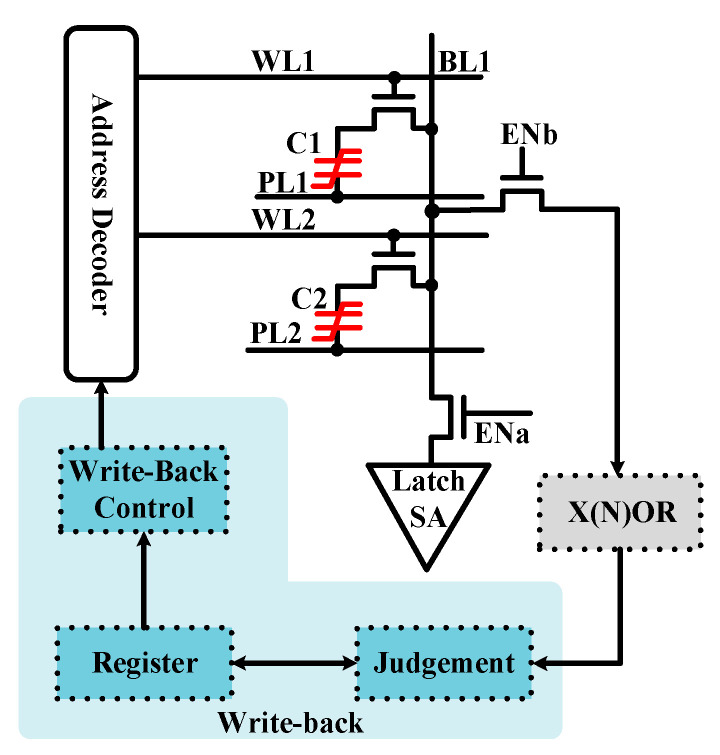
Schematic of the write-back module.

However, for the X(N)OR mode, two rows of data stored on the same BL (or PL) are simultaneously read out, and the data of the cells cannot be written back in time. To solve this issue, the write-back circuits are designed, as shown in Figure 10.

**Figure 11:**
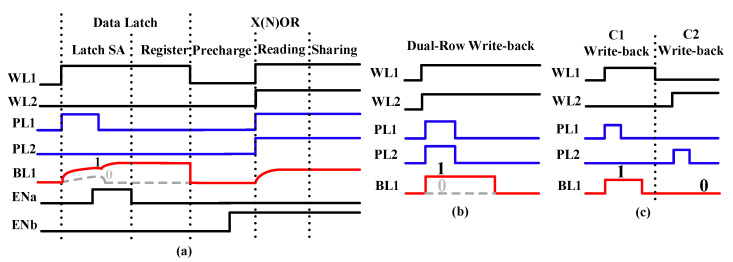
(**a**) The timing of latch data and X(N)OR. (**b**) The timing of write-back when the output of X(N)OR is “0”. (**c**) The timing of write-back when the output of X(N)OR is “1”.

In our previous study [27], the write-back module for the 1T1C-type FeCAP is designed, as shown in Figure 11. The write-back circuit is comprised of a register, a judgment module, and a control module.

The write-back process of the 1T1C-type FeCAP is divided into four phases, as shown in Figure 11. In the first phase, the data in C1 is read out by the latch SA and saved in the register. In the second phase, the X(N)OR function is executed. In the third phase, the data initially stored in C2 is obtained through the judgment module, according to the outputs of X(N)OR and the data in the register. Finally, C1 and C2 are written back. In detail, when the output of X(N)OR is “0”, the data stored in C2 and C1 is written back simultaneously. When the output of X (N)OR is “1”, the data stored C1 is written back first and then C2.

**Figure 12:**
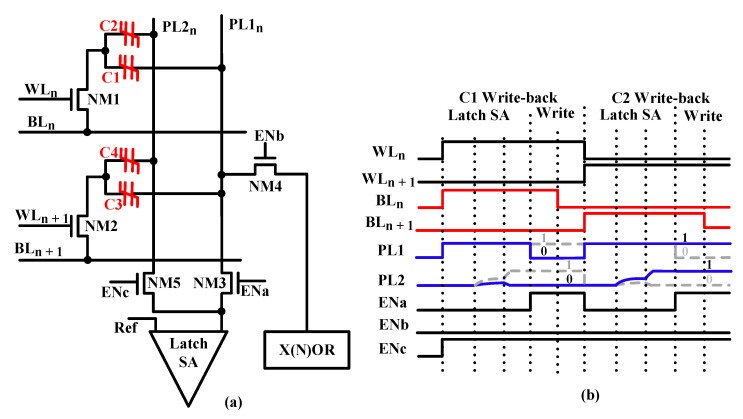
(**a**) The two-step write-back circuit of the 1T2C-type FeCAP. (**b**) Timing of the two-step write-back circuit.

Compared with the 1T1C-type FeCAP, the write-back circuit of the 1T2C-type FeCAP is simpler, as shown in Figure 12a. In the X(N)OR process, data stored in FeCAPs C1 and C3 is sensed. Meanwhile, FeCAPs C2 and C4 keep the original data of C1 and C3.

The write-back process consists of two phases, as shown in Figure 12b. In the first phase, the data in C2 is read out by the latch SA, and the rewriting of the sensed data in C1 and C2 is realized with the assistance of the latch SA. In the second phase, C3 and C4 are written back the same as the write operation discussed in Section 3.1.

The register and decision module are omitted in the proposed 1T2C-type FeCAP write circuit. The two-step write-back circuit can reuse the read-write circuit of the traditional FeCAP without extra circuits, which not only greatly saves the area but also reduces the write-back latency.

## 4. Verification Results and Discussion

X(N)OR logic circuits and the two-step write-back method were simulated with a 28 nm CMOS logic process. The FeCAP model is fitted to the test data by the Landau-Khalatnikov (L-K) equation and then embedded into the simulation tool.

### 4.1. Device Fabrication, Performance, and Simulation Model

**Figure 13:**
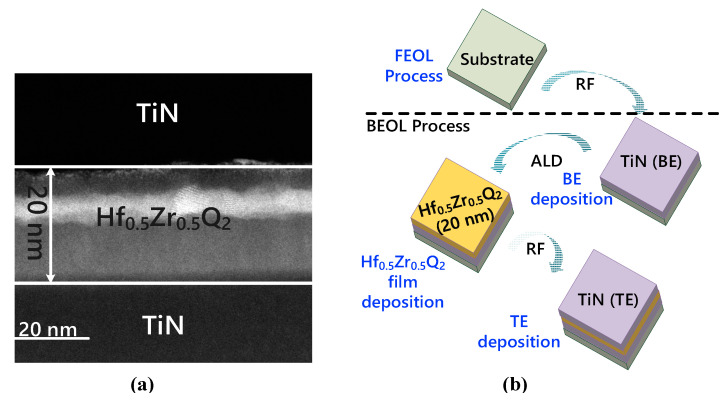
(**a**) Cross-sectional TEM images of a 20 nm thick HfO2 ferroelectric capacitor. (**b**) Fabrication process flow of a Hf_0.5_Zr_0.5_O_2_ ferroelectric capacitor.

The FeCAP used in this study is fabricated using the back-end-of-line (BEOL) process and is fully compatible with the logic process. Figure 13a shows the cross-section transmission electron microscope (TEM) image of the fabricated FeCAP. In the BEOL process, 30-nm TiN was deposited as the bottom electrode (BE) by RF reactive sputtering. Then, 20-nm Hf_0.5_Zr_0.5_O_2_ was deposited on the BE by atomic layer deposition (ALD), where HfO_2_ and ZrO_2_ in Hf_0.5_Zr_0.5_O_2_ are configured in a stoichiometric ratio of 1:1. Finally, 30-nm TiN was deposited as the top electrode (TE) by RF reactive sputtering, and rapid thermal annealing was carried out. The fabrication process flows are shown in Figure 13b.

Previous studies have proved that HfO_2_-based films are very thin with a wide bandgap, as shown in Table 1 [35]. Hence, a Hf_0.5_Zr_0.5_O_2_-based FeCAP maintains good ferroelectricity as the process scales go down.

**Figure 14:**
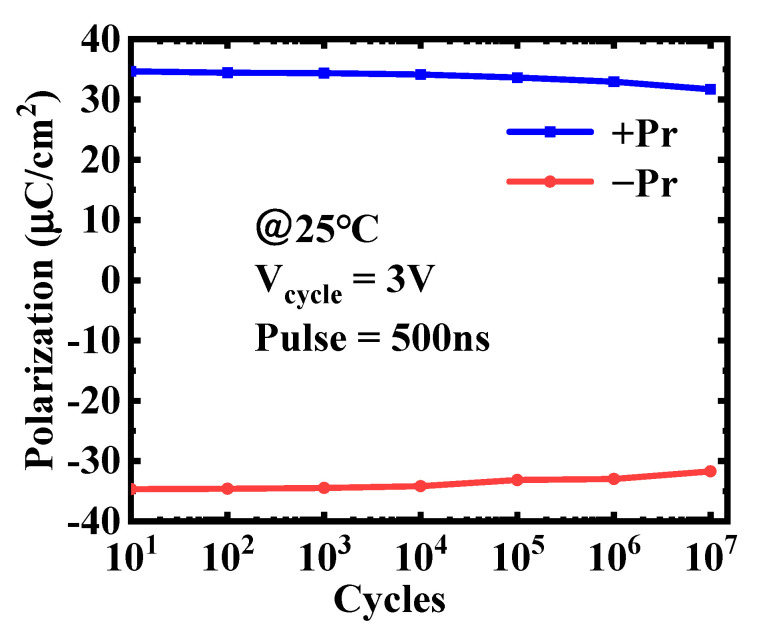
The endurance of the FeCAP used in this work.

Moreover, multiple read-write operations will be performed on the 1T2C-type FeCAP cells to realize the in-memory X(N)OR logic operation. Hence, the endurance of the FeCAP is critical to the function of the X(N)OR circuit. Figure 14 shows the endurance of the FeCAP used in this study. The measurement results show that the FeCAP achieves ~10^7^ stress cycles at 3 V/500 ns pulse at room temperature. In the X(N)OR mode, the FeCAP is biased <3 V and the read–write pulse width is less than 500 ns, so it could achieve much more cycles. Hence, the Hf_0.5_Zr_0.5_O_2_-based FeCAP has sufficient reliability to ensure the efficiency of the X(N)OR logic.

**Figure 15:**
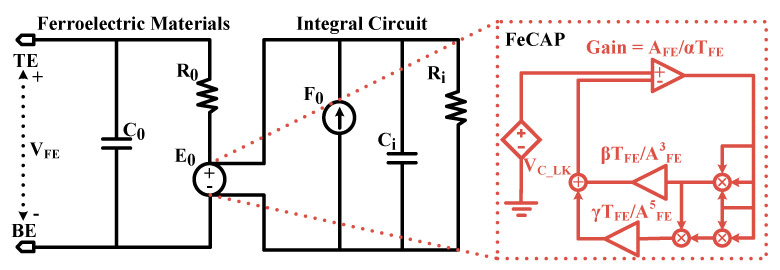
Ferroelectric capacitance model.

The FeCAP model was carried out by the L-K equation proposed by Aziz et al. [36] and optimized based on the measured data. The schematic diagram of the model is shown in Figure 15. A fifth-order polynomial voltage-controlled voltage-source (E_0_) is used to characterize the FeCAP. The polynomial coefficients of E_0_ are derived from the following L-K equation [36, 37]:

(1) $$E-\rho \frac{\mathrm{dp}}{\mathrm{dt}}=\alpha P+{\beta P}^{3}+{\gamma P}^{5}$$

Here, α, β, and γ are the static parameters of the FeCAP and ρ is the kinetic coefficient [37]. Let Q_P_ be the polarization charge stored in the FeCAP, and A_FE_ and T_FE_ are its area and thickness, respectively. Then, the voltage V_FE_ across the FeCAP is:

(2) $$V_{\mathrm{FE}}=\left(\rho \frac{T_{\mathrm{FE}}}{A_{\mathrm{FE}}}\frac{{\mathrm{dQ}}_{P}}{\mathrm{dt}}\right)+\left(T_{\mathrm{EF}}\left\{\frac{{\alpha Q}_{P}}{A_{\mathrm{FE}}}+\frac{{\beta Q}_{P}^{3}}{A_{\mathrm{FE}}^{3}}+\frac{{\gamma Q}_{P}^{5}}{A_{\mathrm{FE}}^{5}}\right\}\right)$$

The FeCAP is modeled as a nonlinear capacitor (C_LK,_ simplified to E_0_) that is connected in series with a resistor (R_0_ = ρT_FE_/A_FE_), which is easy to implement in circuit simulation tool [36], as shown in Figure 15. Table 2 shows the model parameters of the FeCAP in Figure 15, where C_0_ is the parasitic parameter of ferroelectric materials. The current flow in R_0_ and E_0_ is captured through the current-control current-source (F_0_). C_i_ is charged by the current of F_0_. The voltage on E_0_ is equal to the charge on the FeCAP when C_i_ is chosen as 1 F.

**Figure 16:**
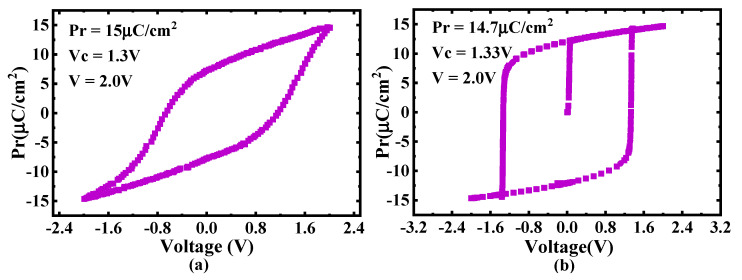
(**a**) Measured and (**b**) simulated P-V curves.

The measured P-V curve of the FeCAP is shown in Figure 16a. The coercive voltage of the FeCAP is about 1.3 V. Hence, when the voltage of BL and PL is 1.8 V, the FeCAP provides a sufficient margin for the proposed mode-switchable SA circuit. The simulation P-V curve is in good agreement with the test results, as shown in Figure 16b.

### 4.2. X(N)OR Logic Operation Simulation Results

The X(N)OR logic operation is simulated and verified using a 28 nm CMOS process. The operating voltage of the enable transistors (NM3 and NM4), as well as the precharge transistors (NM5 and NM10) and inverter (NM9 and PM8) in X(N)OR is 0.9 V of core voltage. The operating voltage of the transmission gate (NM6 and PM7) and selector transistors (NM1 and NM2) is 1.8 V of IO voltage. The VDD in Figure 7 was set to 0.9 V during the simulation.

**Figure 17:**
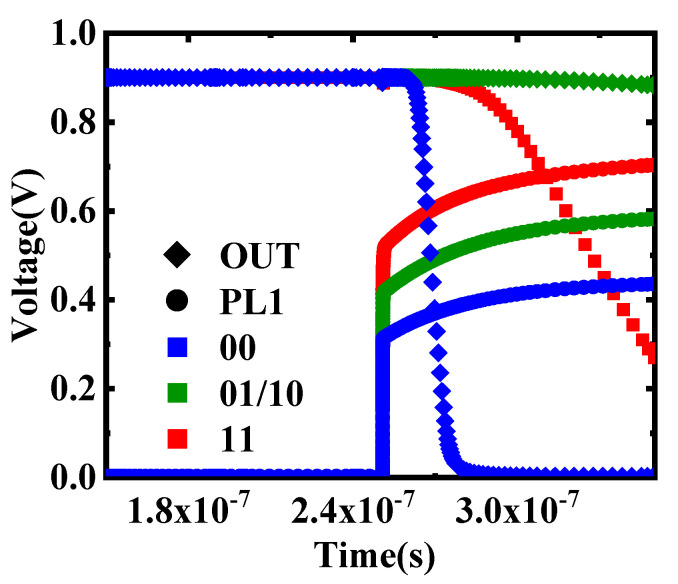
The simulation results of X(N)OR.

When the data in the two 1T2C cells are “01/10”, the output of X(N)OR remains constant at a high level. When the data in the two memory cells are “00”, the output of X(N)OR is pulled down rapidly. When the data in the two memory cells are “11”, the output of X(N)OR is slowly pulled down. Simulation results show that the X(N)OR circuit can work correctly within 100 ns, as shown in Figure 17.

**Figure 18:**
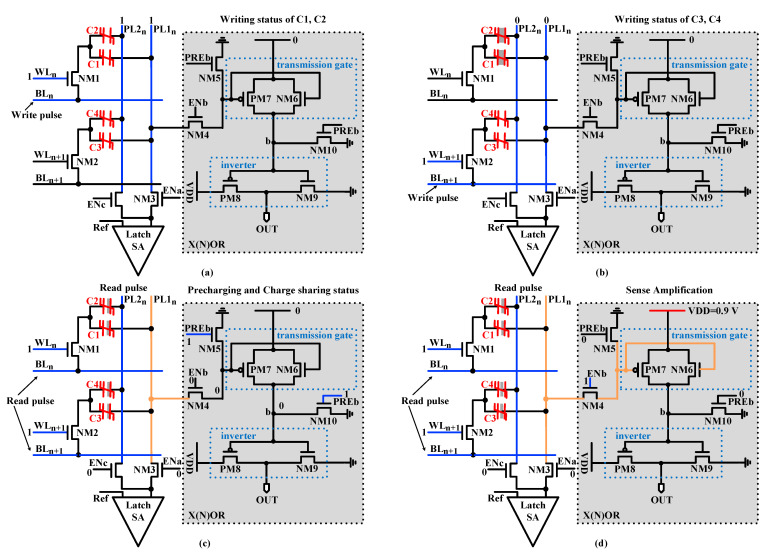
Dual-row activation to realize X(N)OR. (**a**) The writing status of C1 and C2. (**b**) The writing status of C3 and C4. (**c**) Precharging and charge-sharing process. (**d**) X(N)OR readout process.

Figure 18 shows the workflow in detail. During simulation, first, the FeCAPs (C1–C4) in the initial state are polarized; that is, data “0” or “1” is written into C1, C2 and C3, C4 in two steps according to the timing in Figure 5b. These two processes are shown in Figure 18a,b. Second, two word-lines (WLn and WLn+1) are activated simultaneously by an optimized row decoder (ORD), which enables multiple row activation required for bitwise in-memory X(N)OR operations between operands. Third, the charges in C1 and C3 are flushed out, and the voltage of PL1 (V_PL1_) is pulled up by the charge-sharing of FeCAPs. These two processes are shown in Figure 18c. Finally, as shown in Figure 18d, the X(N)OR circuit outputs digital bit “0” or “1” with the help of SA. As shown in Figure 17, when data in the two FeCAPs (C1 and C3) are “00”, V_PL1_ rises to about 400 mV, and PM7 is turned on. The output of X(N)OR is “1”. When data in the two FeCAPs (C1 and C3) are “01” or “10”, V_PL1_ rises to about 550 mV, and PM7 and NM6 are turned off. The output of X(N)OR remains constant at “1”. When data in the two FeCAPs (C1 and C3) are “11”, V_PL1_ rises to about 700 mV, and NM6 is turned on. The output of X(N)OR is “0”. Table 3 shows the truth table of the X(N)OR circuit.

### 4.3. Reliability of In-Memory X(N)OR Logic Operation

**Figure 19:**
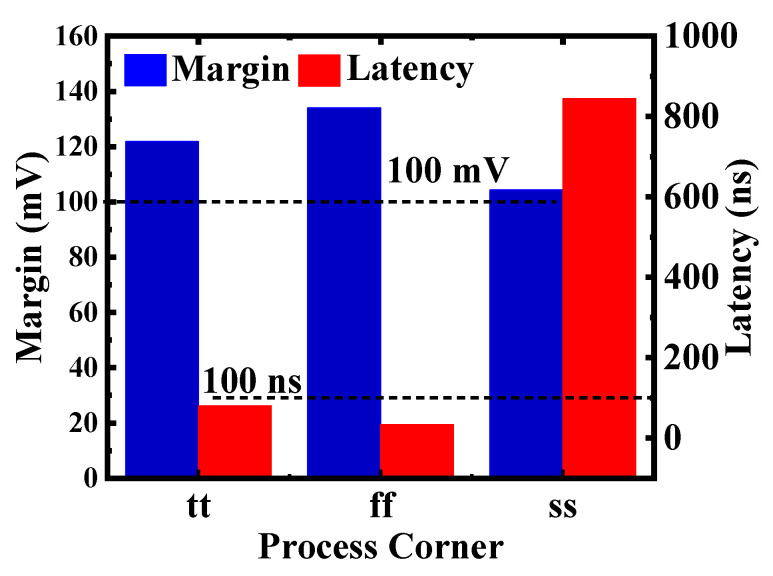
Minimum margin of the neighboring states and maximum latency time under different process corners in the X(N)OR mode.

**Figure 20:**
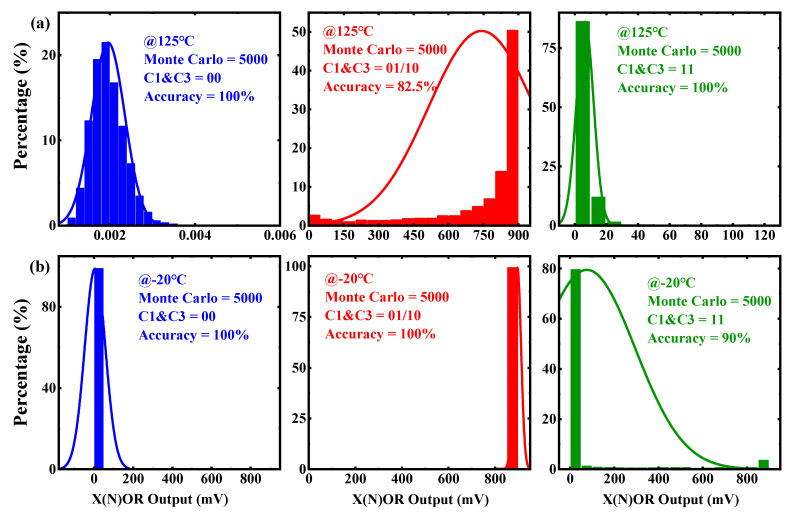
Monte Carlo simulation results of X(N)OR (**a**) at 125 °C (**b**) at −20 °C.

The reliability of the X(N)OR circuit is verified using different simulation conditions. Figure 19 and Figure 20 show the performances of the X(N)OR logic under different process corners. Figure 19 shows that the minimum margins of the neighboring states across different process corners exceed 100 mV, which ensures the circuit can work correctly. Moreover, the reliability of the X(N)OR circuit is also carried out using a 5000-sample Monte Carlo simulation at 125 °C and −20 °C, as shown in Figure 20a,b. The results proved that the X(N)OR logic works even process variations exist. All these show that the performance of the proposed 1T2C FeCAP-based in-memory X(N)OR logic is robust.

## 5. Conclusions

This study proposed a 1T2C FeCAP-based in situ bitwise X(N)OR logic operation scheme. A two-line activation technique was used during the X(N)OR process. Assistant circuits, including an SA and control module, were designed to implement bit-by-bit X(N)OR operations in the same BL. The 1T2C cell structure used in this work can overcome the destructive reading issue of the 1T1C FeCAP cell with a two-step write-back circuit. The circuit was verified in a 28 nm CMOS logic process with the FeCAP model carried out from the L–K equation. Table 4 summarizes the key parameters and compares our work with previous studies. The proposed circuit has the advantages of low design complexity, small area, high memory efficiency, and nonvolatility.

## Figures and Tables

**Table 1:** Comparison of material properties and scalability between HfO_2_, PZT (lead zirconate titanate), and BTO (barium titanate).

Characteristics	PZT	BTO	HfO_2_
Thickness (nm)	>70	>25	5–40
Bandgap (eV)	3–4	~3.1	5.3–5.6
Dielectric constant	~1300	150–250	~30
CMOS compatibility	Pb and O_2_ diffusion	Bi and O_2_ diffusion	Stable
Remnant polarization (2Pr) (μC/cm^2^)	20–40	<10	1–40

**Table 2:** Model parameters of the FeCAP model.

**Model parameter**	α (m/F)	β (m^5^/F/C^2^)	γ (m^9^/F/C^4^)	R_0_ (Ω)	C_0_ (pF)
**Value**	−6.25 × 10^9^	4.88 × 10^27^	1.43 × 10^47^	625	288

**Table 3:** The truth table of the in-memory X(N)OR circuit.

C1	C3	OUT
0	0	0
1	0	1
0	1	1
1	1	0

**Table 4:** Performance comparison of several in-memory X(N)OR logic implementation plans.

Architecture	Nonvolatile	Memory Cell	Technology	X(N)OR-Aera
SRAM-based [16]	No	6T	28 nm	SA and Ref
DRAM-based [26]	No	1T1C	45 nm	10T
RRAM-based [20]	Yes	1T1R	65 nm	CSA and Ref
MRAM-based [21,22]	Yes	2T1MTJ/1MTJ	28 nm/40 nm	12T/15T
1T1C FeCAP-based [27]	Yes	1T1C	28 nm	5T
1T2C FeCAP-based	Yes	1T2C	28 nm	5T

## Data Availability

The data that support the findings of this study are available from the corresponding author upon request.
